# Distribution of interleukin-1 receptor complex at the synaptic membrane driven by interleukin-1β and NMDA stimulation

**DOI:** 10.1186/1742-2094-8-14

**Published:** 2011-02-11

**Authors:** Fabrizio Gardoni, Mariaserena Boraso, Elisa Zianni, Emanuela Corsini, Corrado L Galli, Flaminio Cattabeni, Marina Marinovich, Monica Di Luca, Barbara Viviani

**Affiliations:** 1 Department of Pharmacological Sciences, University of Milan, Via Balzaretti 9, 20133 Milan, Italy

## Abstract

Interleukin-1β (IL-1β) is a pro-inflammatory cytokine that contributes to neuronal injury in various degenerative diseases, and is therefore a potential therapeutic target. It exerts its biological effect by activating the interleukin-1 receptor type I (IL-1RI) and recruiting a signalling core complex consisting of the myeloid differentiation primary response protein 88 (MyD88) and the IL-1R accessory protein (IL-1RAcP). This pathway has been clearly described in the peripheral immune system, but only scattered information is available concerning the molecular composition and distribution of its members in neuronal cells. The findings of this study show that IL-1RI and its accessory proteins MyD88 and IL-1RAcP are differently distributed in the hippocampus and in the subcellular compartments of primary hippocampal neurons. In particular, only IL-1RI is enriched at synaptic sites, where it co-localises with, and binds to the GluN2B subunit of NMDA receptors. Furthermore, treatment with NMDA increases IL-1RI interaction with NMDA receptors, as well as the surface expression and localization of IL-1RI at synaptic membranes. IL-1β also increases IL-1RI levels at synaptic sites, without affecting the total amount of the receptor in the plasma membrane. Our results reveal for the first time the existence of a dynamic and functional interaction between NMDA receptor and IL-1RI systems that could provide a molecular basis for IL-1β as a neuromodulator in physiological and pathological events relying on NMDA receptor activation.

## Findings

Interleukin-1β (IL-1β) is a pro-inflammatory cytokine that is involved in the pathogenesis of a number of neurological disorders, possibly as a modulator of glutamatergic response [1]. This suggestion arises from the observation that IL-1β is often over-produced in injured tissues in which there are high levels of glutamate [2-4], and this over-production has been related to the exacerbation of glutamate-driven pathological conditions [4-6]. Various mechanisms have been identified that may explain the convergence between the IL-1β and glutamatergic systems [1], including hyperactivation of the NMDA receptor (NMDAR). IL-1β increases the activity of hippocampal neuronal NMDARs by phosphorylating the GluN2B subunit and thus enhancing NMDA-induced neuronal death [7]. The same mechanism is recruited in neurons as a consequence of the IL-1β released from glia by the HIV-virus glycoprotein gp120 [8], and underlies the pro-convulsive effect of IL-1β [9]. Whatever mechanism may be recruited by IL-1β, the involvement of IL-1RI is suggested by the uncontested neuroprotective effect of the IL-1 receptor antagonist (IL-1ra) [4,10].

The binding of IL-1β to IL-1RI in the immune system leads to its association with the IL-1R accessory protein (IL-1RAcP) [11] and the myeloid differentiation primary response protein 88 (MyD88) [12] to form *the core *of the IL-1β/IL-1R signalling complex. However, little information is currently available concerning the molecular composition of the members of the IL-1R complex, or their subcellular distribution and functional cross-talk with NMDARs in neuronal cells [13-15]. This is a major gap in our knowledge of the pathological mechanisms involving IL-1β/IL-1RI in neurons that may be relevant to therapeutic interventions in the central nervous system (CNS).

The distribution of IL-1RI, IL-1RAcP and MyD88, together with the pre- and post-synaptic markers synaptophysin and PSD-95, was investigated in different subcellular compartments purified from adult rat hippocampi by means of western blotting [16], and by means of confocal microscopy of primary hippocampal neurons.

**Figure 1:**
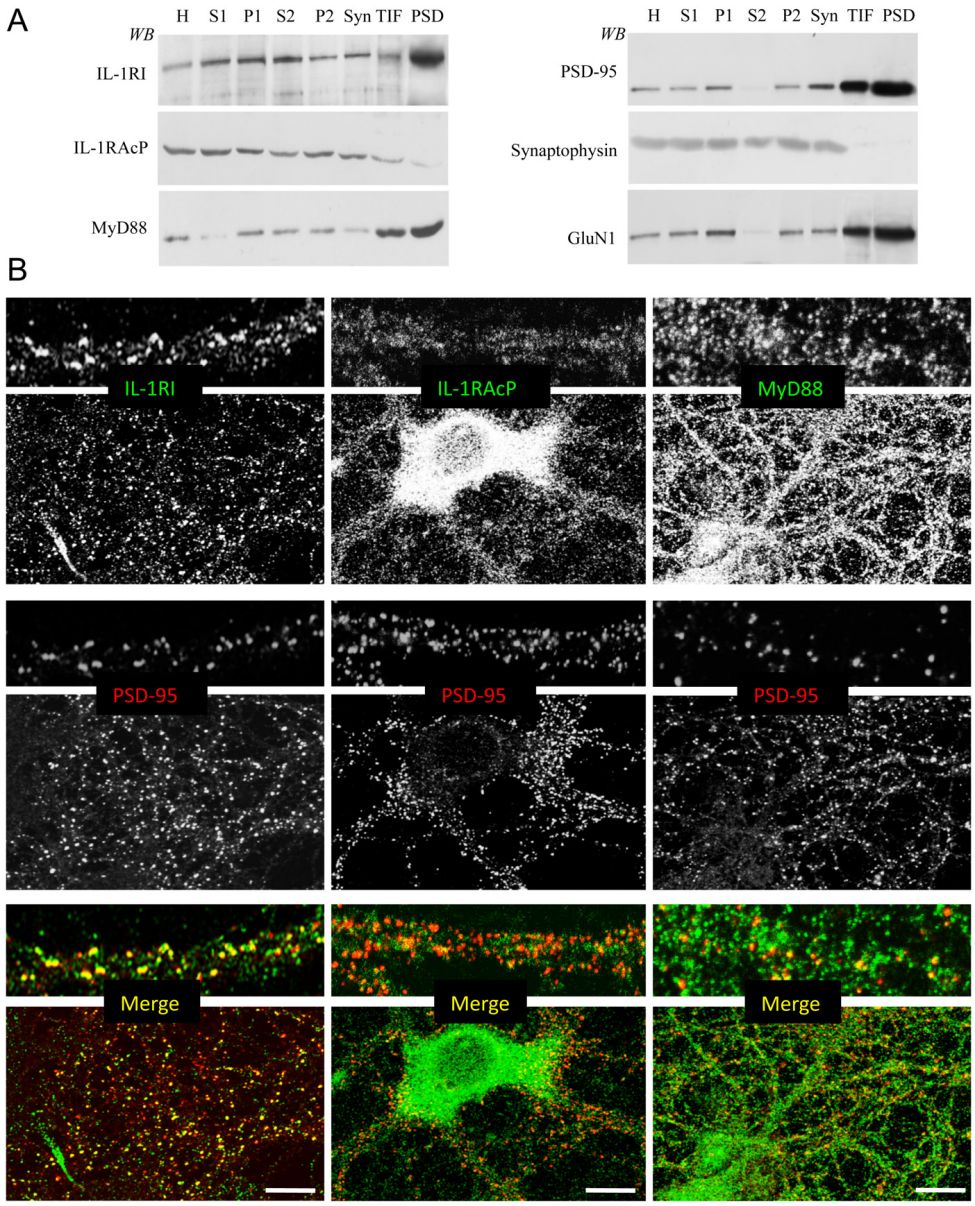
**Characterisation of IL-1RI, IL-1RAcP and MyD88 subcellular distributions in rat hippocampi and primary hippocampal neurons**. **A**: IL-1RI, IL-1RAcP and MyD88, together with markers of the pre-synaptic compartment (synaptophysin) and post-synaptic side (PSD-95, GluN1) were analysed in various rat hippocampus subcellular compartments by means of western blot. H = homogenate; S1 = low-speed supernatant; P1 = nuclei-associated membranes; S2 = high-speed supernatant; P2 = crude membrane fraction; Syn = synaptosomes; TIF = Triton-insoluble postsynaptic fraction; PSD = post-synaptic density. **B**: *DIV14 *hippocampal neurons were immunolabelled for IL-1RI, IL-1RAcP and MyD88 (upper panels), and PSD-95 as a post-synaptic marker (middle panels). The bottom panels show the merged images. Scale bar: 10 μM. High-magnification images are shown at the top of each panel. The antibody specific for IL-1RI was from Santa Cruz Biotechnology, Inc (Santa Cruz, CA) (M20) and its specificity has been tested by pre-absorption with the blocking peptide (data not shown).

Subcellular fractionation showed that IL-1RI, MyD88 and IL-1RAcP were present in all of the tested fractions but, although IL-1RI and MyD88 were particularly enriched in the postsynaptic density (PSD) fraction (Figure 1A, left panel), together with PSD-95 and the GluN1 subunit of the NMDA receptor (Figure 1A, right panel), only traces of IL-1RAcP were present in the postsynaptic Triton-insoluble fraction (TIF) and PSD (Figure 1A, left panel). Confocal imaging showed that IL-1RI is distributed along dendrites and enriched in the post-synaptic compartment, as shown by the high degree of co-localisation with PSD-95 (34.3% ± 3.7%; Figure 1B, left panels). MyD88 was uniformly distributed along the neurons and moderately co-localised with PSD-95 (15.6% ± 2.8%; Figure 1B, right panels). IL-1RAcP labelling was intense and diffuse in the somatic cytoplasm of cultured neurons, and low and diffuse along the dendrites, and hardly co-localised with PSD-95 (4.1% ± 1.9%; Figure 1B, central panels). Overall, these data suggest that there is a different subcellular distribution of the members of the IL-1R complex protein in neurons, with IL-1RI (and, to a lesser extent, MyD88) being enriched at the post-synaptic sites.

**Figure 2:**
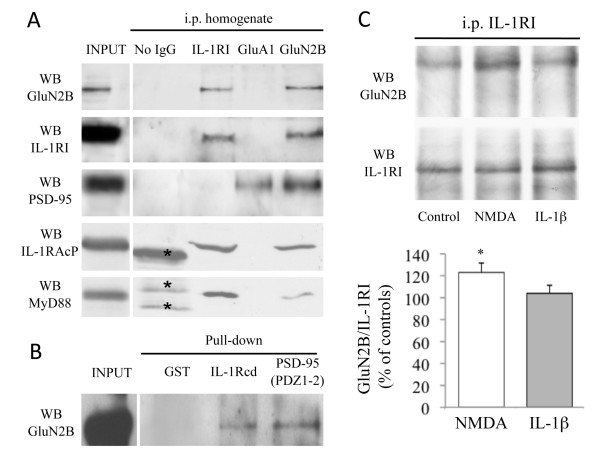
**Interaction between IL-1RI and the GluN2B subunit of NMDA receptors**. **A**: Total homogenate was immunoprecipitated (i.p.) with antibodies against IL-1RI, GluA1 or GluN2B, and the presence of GluN2B, IL-1RI, PSD-95, IL-1RAcP and MyD88 in the immunocomplex was evaluated by means of western blot. IL-1RI, IL-1RAcP and MyD88 co-precipitated with GluN2B but not with GluA1. (*) Nonspecific bands were detected in the No IgG lane. **B**: GST-IL-1R(CD) and GST-PSD-95(PDZ1-2) fusion proteins, and GST alone were incubated in a pull-down assay with total homogenate from rat hippocampus. The western blot analysis was performed using the GluN2B antibody. **C**: Hippocampal cultures were exposed in the absence or the presence of IL-1β (30 minutes, 0.05 ng/ml) or NMDA (10 minutes, 50 μM). Neuronal lystes were immunoprecipitated with anti-IL-1RI, and the presence of GluN2B and IL-1RI in the immunocomplex was evaluated by means of western blot. Treatment with NMDA but not with IL-1β led to a significant increase in the IL-1β/GluN2B complex (p < 0.05 NMDA *vs *control).

Co-immunoprecipitation experiments involving the components of the IL-1R complex and the AMPA and NMDA receptor subunits were performed to assess the localisation and interactions of IL-1RI within distinct subdomains of the PSD structure [17]. Protein homogenates (200 μg) from rat hippocampi were immunoprecipitated [18] with antibodies specific for IL-1RI, for the GluA1 subunit of AMPA receptors, or for the GluN2B subunit of the NMDA receptor. Each sample was then evaluated for the presence of: i) IL-1RI, IL-1RAcP and MyD88; ii) the GluN2B subunit: and iii) PSD-95. Figure 2A shows that, in hippocampal lysates, IL-1RI not only co-precipitated with IL-1RAcP and MyD88, but also with GluN2B; the absence of any PSD-95 signal in the IL-1RI co-immunoprecipitates excludes the possibility that, under our experimental conditions, the co-precipitation of GluN2B reflected nonspecific immunoprecipitation of insoluble synaptic proteins. In line with this, GluN2B co-precipitated with IL-1RI, thus confirming the association between these components (Figure 2A, right lane). Finally, none of the members of the IL-1R complex was detectable in the immunocomplex of the GluA1 subunit of AMPA receptors, which suggests a specific interaction between the GluN2B subunit of the NMDA receptor and IL-1R complexes (Figure 2A).

The association between IL-1RI and GluN2B was confirmed by a pull-down assay based on a fusion protein of the cytoplasmic domain of IL-1RI with GST (GST-IL-1Rcd) (Figure 2B), which contained the C-terminal 369-569 aa domain of IL-1RI. As a positive control, we used a GST-PSD-95 (PDZ1-2) fusion protein that has been previously shown to bind the GluN2B subunit of NMDA receptors [18]. Lysates from rat hippocampal neurons were applied to affinity beads and extensively washed, after which the bound material was resolved by SDS-PAGE and underwent immunoblotting analysis using an antibody raised against GluN2B. Figure 2B shows that both IL-1Rcd and PSD-95 (PDZ1-2) associated with the GluN2B subunit, thus confirming a specific association between IL-1RI and GluN2B.

As it is well known that the synaptic localisation of receptors and ion channels, together with their protein-protein interactions, are modulated in response to various stimuli, and that they undergo dynamic changes under physiological and pathological conditions [19,20], we investigated the possibility that IL-1RI distribution and interaction with GluN2B may be dynamically modulated. Given the relationship between the IL-1β receptor complex and NMDAR, we treated primary hippocampal neurons with IL-1β, 0.05 ng/ml, for 30 min (a concentration that also enhances NMDAR activity) [7] or NMDA, 50 μM, in ACSF buffer [7]: the NMDA was applied to the neurons for 10 min, after which the cells were washed and incubated for a further 20 min in ACSF buffer. We first tested whether IL1-β and/or NMDA modulated the interaction between IL-1RI and the GluN2B subunit of the NMDA receptor (Figure 2C). IL-1RI was immunoprecipitated from total lysates of primary hippocampal neurons treated or not with NMDA, 50 μM, or IL-1β, 0.05 ng/ml, and assayed for GluN2B by means of western blotting (Figure 2C). The results show that only NMDA significantly increased the interaction between IL-1RI and GluN2B (Figure 2C; p < 0.05 NMDA *vs *control).

**Figure 3:**
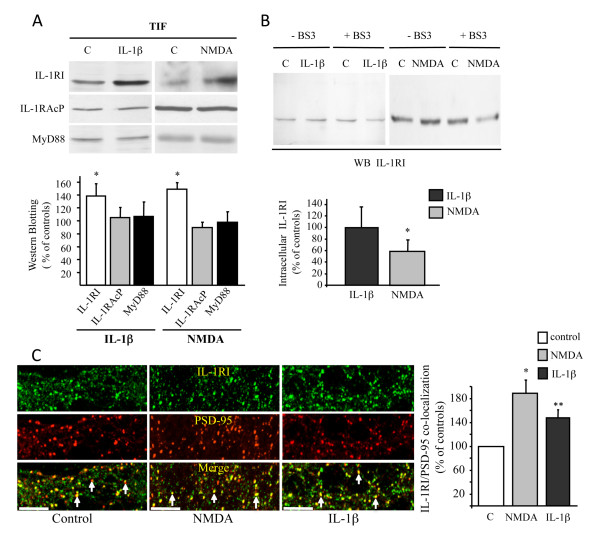
**Effect of NMDA and IL-1β on IL-1RI subcellular localisation**. **A**: Western blot analysis of the TIF fraction obtained from control, IL-1β-treated (0.05 ng/ml) and NMDA-treated hippocampal cultures (50 μM). The same amount of proteins was loaded in each lane. IL-1β increases IL-1RI localization in the Triton-insoluble fraction (TIF) (*p <0.05) leaving unaffected IL-1RAcP and MyD88 levels. Values are means ± S.E of 4 independent experiments. **B**: Western blot of IL-1RI from control, IL-1β-treated (0.05 ng/ml) and NMDA-treated (50 μM) hippocampal cultures exposed (+BS^3 ^lanes) or not (-BS^3 ^lanes) to the cross-linking agent BS^3^. IL-1RI high-molecular-weight complexes that didn't enter the gel are not shown. **C**: Hippocampal neurons were either left untreated (control) or treated with IL-1β (0.05 ng/ml, 30 minutes) or NMDA (50 μM) fixed, and immunolabeled for IL-1RI (green) and PSD-95 (red) as a postsynaptic marker. Data are expressed as percentage of IL-1RI colocalization with PSD-95 (AIM4.2 software, Zeiss). White arrows indicate PSD-95 positive clusters in the merge panel. Scale bar: 5 μM.

We then evaluated whether the members of the IL-1β receptor complex could be re-distributed in different neuronal compartments after stimulation with IL-1β or NMDA. Both NMDA and IL-1β significantly increased the amount of IL-1RI in the postsynaptic TIF fraction (Figure 3A; p < 0.05, IL-1β or NMDA *vs *control). The treatments did not affect the synaptic distribution of either IL-1RAcP or MyD88. These results were confirmed by confocal microscopy (Figure 3C), which showed an increase in the co-localisation of IL-1RI with PSD-95 as quantified in the graph (Figure 3C).

The increase in IL-1RI receptors at the postsynaptic site may be due to new synthesis and delivery of receptors from the endoplasmic reticulum, or to lateral diffusion from adjacent compartments [19,21], and this was addressed by carrying out surface expression assays using the non-cleavable, membrane-impermeable crosslinking agent BS^3 ^[22]. Primary hippocampal neurons were treated with IL-1β, 0.05 ng/ml, or NMDA, 50 μM, and then exposed to BS^3^, lysed and blotted for IL-1RI. The intracellular amount of IL-1RI was reduced by NMDA but not by IL-1β (p < 0.05, NMDA *vs *control; Figure 3B). The reduction in intracellular IL-1RI after NMDA exposure, together with its increase in the synaptic fraction, suggests that NMDAR activation favours the membrane insertion of new IL-1RI. Alternatively, the increase in IL-1RI in the synaptic membrane may be attributable to stabilisation of the complex with NMDAR (within the core of the PSD), which could prevent lateral movement and/or endocytosis. In either case, a new pool of receptors would be made available. On the contrary, IL-1β possibly enriches IL-1RI at post-synaptic sites, promoting its lateral translocation (i.e. membrane diffusion) from extra-synaptic sites; however, this probably does not occur within the core microdomain of the PSD, as suggested by the unchanged levels of IL-1RI associated with the NMDAR complex.

In conclusion, ours are the first findings showing a molecular interaction between IL-1RI and the GluN2B subunit of NMDAR, and suggest a new molecular mechanism by means of which IL-1β and NMDA may dynamically regulate IL-1RI at post-synaptic sites. Furthermore, NMDA-dependent activation increases the amount of IL-1RI inserted into the membrane capable of interacting with released IL-1β. This suggests a new molecular mechanism by means of which IL-1β may contribute to excitotoxicity, thus opening up new possibilities for targeted inhibition strategies that can be used in IL-1β/glutamate-driven CNS diseases.

## Abbreviations

IL-1β: interleukin-1β; IL-1ra: IL-1 receptor antagonist; IL-1RI: interleukin-1 receptor type I; IL-1RAcP: IL-1R accessory protein; MyD88: myeloid differentiation primary response protein 88; NMDAR: NMDA receptor; PSD: postsynaptic density; TIF: Triton-insoluble postsynaptic fraction.

## Competing interests

The authors declare that they have no competing interests.

## Authors' contributions

FG, BV, MDL, MM, EC, FC, and CLG designed the study; FG, MB, EZ and BV performed it; FG, BV and EC contributed new reagents/analytical tools; MB and EZ analysed the data; and FG and BV wrote the paper. All authors have read and approved the final version of the manuscript.
